# Our Contemporaries

**Published:** 1915-06

**Authors:** 


### OUR CONTEMPORARIES.

Le Progres Medical resumed publication with the March issue. Most of the French journals which suspended publication after the July or August issue on account of the mobilization of editorial and printing forces, have resumed.

The Post Graduate has been reduced to a bulletin of the college and hospital.

